# Epidemic Dynamics via Wavelet Theory and Machine Learning with Applications to Covid-19

**DOI:** 10.3390/biology9120477

**Published:** 2020-12-18

**Authors:** Tô Tat Dat, Protin Frédéric, Nguyen T. T. Hang, Martel Jules, Nguyen Duc Thang, Charles Piffault, Rodríguez Willy, Figueroa Susely, Hông Vân Lê, Wilderich Tuschmann, Nguyen Tien Zung

**Affiliations:** 1 Centre de Mathématiques Laurent-Schwartz, École Polytechnique Cour Vaneau, 91120 Palaiseau, France; 2 Torus Actions SAS, 3 Avenue Didier Daurat, 31400 Toulouse, France; protin@torus-actions.fr (P.F.); hangntt@torus-actions.fr (N.T.T.H.); jules@torus-actions.fr (M.J.); ndthang@torus-actions.fr (N.D.T.); charles.piffault@torus-actions.fr (C.P.); fsusely@torus-actions.fr (F.S.); 3 Ecole Nationale de l’Aviation Civile, 7 Avenue Edouard Belin, 31400 Toulouse, France; willy.rodriguez@enac.fr; 4 Institute of Mathematics of the Czech Academy of Sciences, Zitna 25, 11567 Praha 1, Czech Republic; hvle@math.cas.cz; 5 Fakultät für Mathematik, Karlsruher Institut für Technologie (KIT), Englerstr. 2, D-76131 Karlsruhe, Germany; tuschmann@kit.edu; 6 Institut de Mathematiques de Toulouse, Université Toulouse 3, 18 Route de Narbonne, 31400 Toulouse, France; tienzung@math.univ-toulouse.fr

**Keywords:** Covid-19, SARS-CoV-2, epidemic-fitted wavelet, epidemic dynamics, model selection, curve fitting, Covid-19 spread predicting

## Abstract

**Simple Summary:**

Using tools from both mathematics (especially wavelet theory) and computer science (machine learning), we present a general new method for modelling the evolution of epidemics which is not restricted to human populations. A crucial novel feature of our approach is that it significantly takes into account that an epidemic may take place in certain types of waves which cannot only be of a global as well as local nature, but can also occur at multiple different times and locations. In the particular case of the current Covid-19 pandemic, based on recent figures from the Johns Hopkins database we apply our model to France, Germany, Italy, the Czech Republic, as well as the US federal states New York and Florida, and compare it and its predictions to established as well as other recently developed forecasting methods and techniques.

**Abstract:**

We introduce the concept of epidemic-fitted wavelets which comprise, in particular, as special cases the number I(t) of infectious individuals at time *t* in classical SIR models and their derivatives. We present a novel method for modelling epidemic dynamics by a model selection method using wavelet theory and, for its applications, machine learning-based curve fitting techniques. Our universal models are functions that are finite linear combinations of epidemic-fitted wavelets. We apply our method by modelling and forecasting, based on the Johns Hopkins University dataset, the spread of the current Covid-19 (SARS-CoV-2) epidemic in France, Germany, Italy and the Czech Republic, as well as in the US federal states New York and Florida.

## 1. Introduction

The present work proposes a novel method for modelling epidemic dynamics by combining wavelet theory and data-driven model section techniques in machine learning.

In understanding epidemic diffusion and the growth rate of an infectious disease at population level, the actual number of reported cases of infections always plays a (if not *the*) crucial role and, far beyond that, at least in the case of diseases afflicting human societies, directly influences government and health care system decisions and measures regarding, e.g., protection, containment and hospital capacities. However, due to both the manifold practical as well as conceptual issues involved, a rigorous and accurate detection of this number turns out to be a rather difficult and complex problem.

To illustrate at least some of the theoretical difficulties involved here by a prominent and important case which calls the entire world now to action, let us note that most current mathematical modelling and forecasting techniques for the spread of the Covid-19 disease are based on classical Susceptible–Infectious–Recovered/Removed (SIR) and Susceptible-Exposed- Infectious-Recovered/Removed (SEIR) compartmental epidemiological models [1,2,3]. Yet, with regard to predicting the number of infectious cases I(t) at time *t*, they suffer from severe and model-inherent principal limitations:

All these models, as well as all their derivatives, are not suitable to build a model for the function I(t) which is compatible with any given population. This is because these models are based on the assumption that the population is homogeneously composed and distributed (i.e., the chance that an arbitrary infected person will infect an arbitrary susceptible person is taken to be constant throughout the epidemic, and, moreover, it is assumed that at any given time every infected person has one and the same constant chance to recover).

In real life, however, there are actually many and rather diverse waves of outbreaks, stemming from different times or locations. One faces here not only drastically varying growth rates, but also hot spots versus no-cluster locations, infection rates depending on age or other parameters, etc. which altogether entails that the homogeneity assumption approach taken in SIR models and their variations is oversimplified and cannot give realistic forecasts.

To overcome the drawbacks caused by homogeneity assumptions, the new approach presented in this work is based on the following idea: we shall decompose the growth curve of infection numbers into several basic “waves”, where each basic wave is considered as a representation of the epidemic, and localised both in time and position.

This point of view naturally calls for the use of wavelet theory. Wavelets as such are special families of functions which came up in the 1980s by combining older concepts from mathematics, computer science, electrical engineering and physics, having since found fruitful applications in many other disciplines. In particular, some precursor, wave-based approaches to modelling epidemic growth appeared already a long time before wavelets emerged in both deterministic and stochastic models, compare, among others, the works in [4,5,6,7], and only very recently, Krantz et al. (compare with the work in [8]). Moreover, the latter work has also proposed building epidemic growth models by combining wavelet with discrete graph theory (see also below).

In this article, we propose an approach to epidemic dynamics by modelling the number of daily reported cases using specially designed wavelets, called epidemic-fitted (EF) wavelets. For instance, the number I(t) of infectious individuals at time *t* in the classical SIR and SEIR models is an EF wavelet, see Section 3.4. Another example of an EF wavelet is the log-normal one, which we will use in our Covid-19 spread forecasting applications, see Section 3.4 and Section 4.1 for more details.

In our approach, the number of daily reported cases is the value of a function that is a positive linear combination of *N* EF wavelets at the given day. We fix the number *N* of summands of EF wavelets entering in our modelling function (and in our applications *N* is usually taken to be 3 or 5). The wavelet series coefficients themselves are then obtained by machine learning-based curve fitting methods with square loss function, see Section 2.2 and Section 4.1.

We then proceed with specific applications to Covid-19 scenarios. Here, we present, now using in addition data-driven machine learning-based curve fitting, some of our model’s predictions to selected countries and US federal states, which are based on the currently existing respective data for these locations provided by the most recent numbers supplied by the Johns Hopkins University Covid-19 database.

Before mentioning and commenting upon other related works, let us adopt from now on, and throughout all following parts of the present work, the following *convention:* as we shall consider only reported cases in our paper, we will omit the adjective “reported” from “reported cases of infected”. In [9], the authors present three basic “macroscopic” models to fit data emerging from local and national governments: exponential growth, self-exciting branching process and compartmental models. The compartmental models are the classical SIR and SEIR models; the self-exciting branching process has been used before with regard to treating Ebola disease outbreaks and other dynamics of social interaction. In the exponential growth model, the number I(t) of infectious individuals at time *t* is expressed as $I\left(t\right)=I_{0}e^{\alpha t}$, where α is the rate constant. The exponential growth model is related to our approach, in which the exponential function is modelling the reported infections. However, as this is a one-parameter model, it works only well for fitting the data at the beginning of an outbreak.

In [10], the authors use a log-normal density function with three parameters to fit the daily reported cases. However, as they tried to fit the data with only one function, the curve of reported cases may not be well fitted, as there are usually several waves of the epidemic for a period while one function presents only one wave. As explained above, our wavelet approach does overcome this difficulty. In [11], the authors use the function $f\left(x\right)=k\gamma \beta \alpha^{\beta }x^{-1-\beta }\exp\left(-\gamma {\left(\alpha /x\right)}^{\beta }\right)$ with parameters α,β,γ,k to fit all data. This method, too, can fit the data only for one wave. In [12], the authors fit the data of daily reported cases with a two-wave model, using the sum of two Gaussian functions.

In [13], the authors introduce an epidemic model composed of overlapping sub-epidemic waves, where each wave is a generalised logistic growth model given by solution of differential equations. A short-term forecast of the Covid-19 epidemic in China from 5 to 24 February 2020 was given in [14] using three phenomenological models (generalised logistic growth model, the Richards growth model and sub-epidemic wave model in [13]) and ensemble methods (see also [15] for the ensemble approach in forecasting epidemic trajectories). In [16], a multi-wave model combining several SIR models, namely, a Multiple-Wave Forced-SIR model, was introduced to fit the data of daily cases.

Recently, Krantz et al. [8] have proposed an approach to construct epidemic growth models using *fractional* wavelets. These are built from the number of reported cases to construct wavelets that model the dynamics of the number of completed cases [8]. In their paper, the number of completed cases is the sum of the number of reported cases and the number of unreported cases. Furthermore, the proposed approach there is to update their models assuming the availability of the reporting error which improves over time and tends to zero eventually. This assumption appears to us, however, as a too idealistic one.

Those two last approaches are the ones which are most closely related to our own. However, while those use single waves coming from solutions differential equations, we use general wavelet functions such as Gaussian functions, log-normal functions, Gompertz density functions and Beta prime density functions, which all satisfy our general condition of being epidemic-fitted in the sense of Definition 2. We also refer to the works in [17,18,19,20,21,22,23,24,25,26,27,28,29,30,31,32,33,34,35,36,37,38,39,40,41] for other approaches on modelling and forecasting the spread of Covid-19 epidemic using deep learning, machine learning, time series analysis, network model, stochastic model and deterministic compartmental framework.

The remaining parts of the present paper are organised as follows. In Section 2, we first recall the notion of a wavelet (Definition 1) and the fundamental theorem of wavelet theory (Theorem 1), which we are going to put to use in the sequel. We proceed by introducing the notion of an epidemic-fitted (EF) wavelet (Definition 2) and propose our method for modelling epidemic dynamics (Proposition 1), justified by the fundamental Theorem 1. In Section 3, we consider several important examples of EF wavelets and impose constraints on an EF wavelet to be suitable as a basic EF wavelet in epidemic dynamics. In Section 4, we present applications of our method to modelling and forecasting the current spread of Covid-19 in France, Germany, Italy, the Czech Republic and several US federal states, all based on the most recent JHU data.

## 2. Epidemic Modelling via Wavelet Theory and Machine Learning

### 2.1. Wavelets

In this subsection, we recall and collect some basic concepts and facts from Wavelet Theory (cf. [42,43,44]), which will be needed in our approach for modelling epidemic dynamics.

**Definition** **1**  ([42] [p. 24]). *A wavelet or mother wavelet is a function $\psi \in L^{1}\left(R\right)$ such that the following admissibility condition holds:*

(1) $$C_{\psi }=\int_{-\infty }^{\infty }{\left|\hat{\psi }\left(\xi \right)\right|}^{2}\frac{d\xi }{\left|\xi \right|}<\infty ,$$

*where $\hat{\psi }$ is the Fourier transform of ψ, i.e., $\hat{\psi }\left(\xi \right)=\int_{R}\psi \left(x\right)e^{-i\xi x}dx.$*

Notice that condition (1) is only satisfied if $\hat{\psi }\left(0\right)=0$ or ∫ψ(x)dx=0. Conversely, we have the following sufficient condition for (1).

**Lemma** **1**  ([42] [p. 24). *] Let $\psi \in L^{1}\left(R\right)$ and $\int_{R}\psi \left(x\right)dx=0$. If $\int_{R}|\psi \left(x\right)|(1+{\left|x|\right)}^{\alpha }dx<\infty$ for some α>0, then $|\hat{\psi }{\left(\xi \right)|\leq C|\xi |}^{\min(\alpha ,1)}$ and $C_{\psi }<\infty$.*

A basic example of a wavelet is the function

$$\psi \left(t\right)=\frac{\sin(2\pi t)-sin(\pi t)}{\pi t}.$$

From a mother wavelet one can generate other wavelets (called *children wavelets*), using affine transformations (i.e., dilations and translations):

$$\psi_{a,b}\left(t\right)=\frac{1}{\sqrt{\left|a\right|}}\psi \left(\frac{t-b}{a}\right),\;\left(a,b\right)\in R\times R.$$

These wavelets provide us with the following decomposition of $L^{2}\left(R\right)$.

**Theorem** **1**  ([42] [Proposition 2.4.1 and pp. 25–26]). *Let ψ be a mother wavelet. Then, any $f\in L^{2}\left(R\right)$ decomposes as*

(2) $$f=C_{\psi }^{-1}\int_{R^{2}}<f,\psi_{a,b}>\psi_{a,b}\frac{dadb}{a^{2}},$$

*strongly in $L^{2}\left(R\right)$, where <,> denotes the standard scalar product in $L^{2}\left(R\right)$, i.e.,*

(3) $$\lim_{A_{1},A_{2},B\to \infty }{∥f-C_{\psi }^{-1}\int_{1/A_{1}\leq \left|a\right|\leq A_{2},\left|b\right|\leq B}<f,\psi_{a,b}>\psi_{a,b}\frac{dadb}{a^{2}}∥}_{L^{2}}=0.$$

Any function $f\in L^{2}\left(R\right)$ can then be written as a superposition of $\psi_{a_{k},b_{\ell }}$, i.e.,

$$f\left(x\right)=\sum_{k,l}\alpha_{k,\ell }\psi_{a_{k},b_{\ell }}\left(x\right).$$

We refer to the work in [42] for more details on the analysis of discrete wavelet decomposition and, especially, for precise formulas for the coefficients $\alpha_{k,\ell }$.

Notice that from a machine learning point of view, finding the $\alpha_{k,\ell },a_{k},b_{\ell }$ can be thought of as a curve fitting problem, and this is how we will combine wavelet theory and machine learning techniques in our approach to modelling epidemic dynamics.

### 2.2. Epidemic-Fitted Wavelets and Modelling

As we already explained in the introduction, the time development of an epidemic features local as well as global wave-type phenomena. This leads us to the concept of epidemic-fitted wavelets. Informally speaking, such a wavelet is given by a positive real function $W:R\to R^{>0}$, whose value W(t) at a given time *t* describes the number of new infected cases in a homogeneous population with respect to an epidemic that occurs in one wave only, and thus will satisfy some sort of homogeneous compartmental model (without network structure).

As we are interested in the daily infected cases, we can assume that W(t) is strictly positive but tends to 0 when *t* tends to ±∞. Setting w(t)=lnW(t) so that $W\left(t\right)=e^{w(t)}$, the (multiplicative) growth rate of *W* is its log-derivative:

$$\frac{\dot{W}\left(t\right)}{W}=\dot{w}\left(t\right).$$

We wish W(t) to “start” at t=a, (reach its) “peak” at t=χ, and “stop” at t=b (a<χ<b). This is to say that w(a)=w(b)=0, $\dot{w}\left(\chi \right)=0$, $\dot{w}\left(t\right)>0$ for t<χ and $\dot{w}\left(t\right)<0$ for t>χ.

**Definition** **2.**  *Given an interval (a,b)⊂R, a≥0, an epidemic-fitted wavelet is a positive real function $\psi \in L^{1}\left(\left(a,b\right),R^{+}\right)$ such that ψ has start-peak-stop behaviour, i.e., ψ satisfies $\lim_{x\to a^{+}}\psi \left(x\right)=\lim_{x\to b^{-}}\psi \left(x\right)=0$, and ψ admits its maximum at some point in (a,b).*

We can interpret ψ as a wavelet $\tilde{\psi }$ in the sense of Definition 1 by simply setting $\tilde{\psi }\left(x\right):=\psi \left(x\right)$ for x∈(a,b), $\tilde{\psi }\left(x\right):=-\psi \left(|x|\right)$ for x<−a, and ψ(x)=0 otherwise. Indeed, this definition implies that $\int_{R}\tilde{\psi }\left(x\right)dx=0$ and $\int_{R}|\tilde{\psi }\left(x\right)|(1+|x|)dx<\infty$, therefore $C_{\tilde{\psi }}<\infty$ by Lemma 1 and $\tilde{\psi }$ is a wavelet.

The first examples of EF wavelets which come to mind are polynomial functions of degree 3 (restricted to some finite interval). Other examples of functions with start-peak-stop behaviour are Gaussian functions, log-normal functions, Gompertz density functions

(4) $$\psi_{b,c}\left(x\right)=bc\exp\left(c+bx-ce^{bx}\right),$$

and, in SIR models, the solution function giving the number of I(t), the number of infectious individuals (cf. the work in [45], etc.).

In our applications to real data (see Section 4), we will employ log-normal functions as epidemic-fitted (EF) wavelets. For treating an epidemic, we will concentrate on the curve of daily (reported) infected cases, denoted by RC(t), and try to understand the epidemic growth based on this information. Theorem 1 implies that our following ansatz is “asymptotically” correct, as the number *N* grows to infinity. In particular, numerical simulations involving bigger and bigger numbers *N* will lead to better and better accuracy.

**Proposition** **1**  (Ansatz). *A positive function (or curve) whose value is the number of infected cases at time t is representable as a finite linear combination of epidemic-fitted wavelets:*

(5) $$RC\left(t\right)=\sum_{i=1}^{N}\alpha_{i}W_{i}\left(t,\theta_{i}\right),$$

*where each such wavelet $W_{i}$ can be obtained from a basic (mother) EF wavelet ψ by adding some parameters $\theta_{i}=\left(\theta_{i}^{1},\ldots ,\theta_{i}^{k}\right)$.*

Using this ansatz, we shall model epidemic dynamics by finding the wavelet series coefficients $\alpha_{i}$ and $\theta_{i}$ in the decomposition (5), when given the number of infected cases over a sufficient long time frame. This amounts to solving a *curve fitting problem* in machine learning.

## 3. Epidemic-Fitted (EF) Wavelets

In this section, we introduce some epidemic models with different basic (mother) epidemic-fitted (EF) wavelets. In Section 4, we show by fitting the Covid-19 data that log-normal EF wavelet models are highly compatible with the data and lead to very good forecast projections.

### 3.1. Gaussian EF Wavelets

The standard Gaussian function is a fundamental example of a function which has start-peak-stop behaviour and exponential growth:

$$\begin{array}{ccc} \psi :R^{+} & \to & \left(0,1\right]\\ x & \mapsto & \exp(-x^{2}/2). \end{array}$$

After dilating and translating, we obtain a general Gaussian function

$$\psi_{b,c}\left(x\right)=\exp\left(-\frac{{\left(x-b\right)}^{2}}{2c^{2}}\right).$$

We remark that, in general, $\lim_{x\to -\infty }\psi_{b,c}\left(x\right)=0$, but for certain b,c>0 we have $\psi_{b,c}\left(0\right)\ll 1$. In this case, we can simply set $\tilde{\psi }\left(x\right)=\max\left(\psi_{b,c}\left(x\right)-\psi_{b,c}\left(0\right),0\right)$ as the corresponding Gaussian EF wavelet.

In [12], the authors fitted the data of daily reported cases with a two-wave model using the sum of two Gaussian functions. However, as these are symmetric with respect to the the vertical line x=b, this model may be not compatible with the curve of daily cases. We will explain this point in further detail in the next section.

### 3.2. Log-Normal EF Wavelets

We define here the log-normal function, which is a Gaussian function in which the variable *x* is interchanged by logx:

$$\begin{array}{ccc} \psi_{b,c}:R^{+} & \to & \left(0,1\right]\\ x & \mapsto & \exp(-\frac{{\left(\log x-b\right)}^{2}}{2c^{2}}). \end{array}$$

We then define the corresponding log-normal wavelet by extending

$$\psi_{b,c}\left(x\right)=-\exp\left(-\frac{{\left(\log\left(-x\right)-b\right)}^{2}}{2c^{2}}\right),\;\;\mathrm{for}x<0.$$

Thus, we can rewrite it as

$$\psi_{b,c}\left(x\right)=sgn\left(x\right)\exp\left(-\frac{{\left(\frac{1}{2}\log\left(x^{2}\right)-b\right)}^{2}}{2c^{2}}\right).$$

By dilating and translating, we obtain a general log-normal EF wavelet

$$\psi_{b,c,d}\left(x\right)=\exp\left(-\frac{{\left(\frac{1}{2}\log{\left(x-d\right)}^{2}-b\right)}^{2}}{2c^{2}}\right).$$

**Figure 1:**
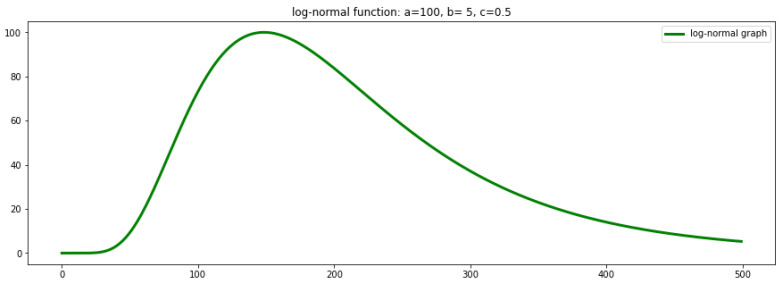
Log-normal graph.

Figure 1 depicts the graph of the log-normal function with scaling coefficient

$$\psi \left(x\right)=a\exp\left(-\frac{{\left(\log x-b\right)}^{2}}{2c^{2}}\right),x>0.$$

### 3.3. Further Examples of EF Wavelets

Based on probability distributions, we can also choose many other functions to build a basic EF wavelet. For example, one can start here from Gompertz density functions

(6) $$\psi_{b,c}\left(x\right)=bc\exp\left(c+bx-ce^{bx}\right),$$

or Beta prime density functions

(7) $$\psi_{b,c}\left(x\right)=x^{b-1}{\left(1+x\right)}^{-b-c}/B\left(b,c\right),$$

where *B* is the Beta function. For appropriately chosen parameters b,c, they all satisfy the epidemic-fitted condition in Definition 2.

Another important class of EF wavelets is given by the function reporting the number of infectious individuals I(t) in compartmental SIR models and their variations (such as SEIR and SIRD models, etc.). The SIR (compartmental) model was introduced by W. O. Kermack and A. G. McKendrick [2], in which they considered a fixed population with only three compartments, and the numbers S(t) (for “susceptible”), I(t) (for “infectious”), and R(t) (for “recovered” (or “removed”)).

(8) $$\begin{array}{ccc} \frac{dS}{dt} & = & -\frac{\beta IS}{N} \end{array}$$

(9) $$\begin{array}{ccc} \frac{dI}{dt} & = & \frac{\beta IS}{N}-\gamma I \end{array}$$

(10) $$\begin{array}{ccc} \frac{dR}{dt} & = & \gamma I. \end{array}$$

**Figure 2:**
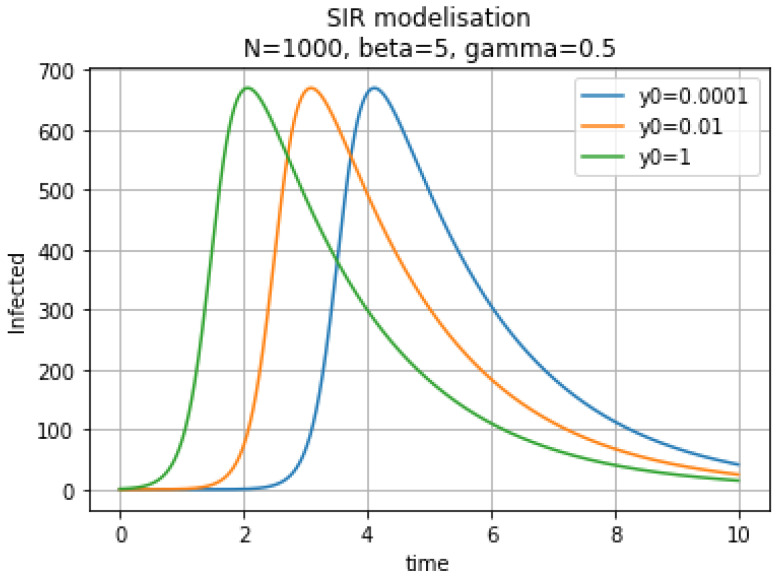
Infectious individuals I(t) for different initial conditions.

In Figure 2, these curves show the number of infectious individuals I(t).

In general, I(t) is an implicit function defined by a system of differential equations, which can lead to difficulties when trying to fit the data. However, we can use here the implicit solutions for simple SIR models which were deduced recently in [45].

### 3.4. Choosing Suitable EF Wavelets

We explain here how to choose good EF wavelets for building an epidemic model. The first criterion to meet is the start-peak-stop behaviour as discussed in Section 2. Our second criterion is based on the following analysis of the number I(t) of infectious individuals in the SIR model:

(11) $$\begin{array}{ccc} \frac{dS}{dt} & = & -\frac{\beta IS}{N} \end{array}$$

(12) $$\begin{array}{ccc} \frac{dI}{dt} & = & \frac{\beta IS}{N}-\gamma I \end{array}$$

(13) $$\begin{array}{ccc} \frac{dR}{dt} & = & \gamma I. \end{array}$$

A closer look at SIR models reveals that the number S(t) of susceptible individuals is decreasing in time. Therefore, the number I(t) of infectious also grows less and the rate of infectious, i.e., dI/dt, before the peak is always less than the one after the peak. This is an important criterion when choosing EF wavelets.

*Log-normal EF wavelets actually turn out to be very good candidates in this regard.* Indeed, the first advantage here is the start-peak-stop behaviour, where the start for a log-normal wavelet is at x=0 (or near 0), the peak is achieved at $x=e^{b}$ and the stop depends on the constant *c*. The second advantage is that at the same value of ψ, the rate of the curve before the peak is less than the one after the peak. This can be easily seen as follows. The derivative of $\psi_{b,c}$ is

(14) $$\psi_{b,c}^{\prime }\left(x\right)=\psi_{b,c}\left(x\right)\frac{-(\log x-b)}{c^{2}x}.$$

Now, suppose that $\psi \left(x_{1}\right)=\psi \left(x_{2}\right)$ with $x_{1}<e^{b}<x_{2}$, then $|\log x_{1}-b|=|\log x_{2}-b|$. Therefore, we have

$$|\psi_{b,c}^{\prime }\left(x_{1}\right)|=\frac{x_{2}}{x_{1}}|\psi_{b,c}^{\prime }\left(x_{2}\right)\left|<\right|\psi_{b,c}^{\prime }\left(x_{2}\right)|$$

as required.

These are the main reasons why we first chose log normal functions as basic EF wavelets for our numerical simulations (see Section 4). We also remark that in [10] the authors used the log-normal density function, i.e., $f_{a,b,c}\left(x\right)=\frac{a}{\sqrt{2\pi }cx}\psi_{b,c}\left(x\right)$, to fit the number of daily reported cases. However, as they used only one single function, and as there are in general many waves of the epidemic, the data may not be well-fitted enough to produce realistic projections.

## 4. Data-Driven Numerical Forecasts

In this section, using log-normal EF wavelets we provide numerical results on the fitting and forecasting of daily new cases of Covid-19 epidemic for some European countries and US federal states.

### 4.1. The Log-Normal Wavelet Model

Our EF wavelet model for the curve of daily new cases is a finite representation by log-normal EF wavelest introduced in Section 4.1:

$$W\left(t\right)=\sum_{i=1}^{N}a_{i}\psi_{b_{i},c_{i}}\left(t\right),$$

where $a_{i},b_{i},c_{i}$ are parameters, *N* is the number of log-normal EF wavelets and *t* is the time variable.

We intend to find the parameters $a_{i},b_{i},c_{i}$ such that W(t) is close to the number of daily infections RC(t) by a suitable loss function L(·,·). In other words, we want to find parameters which minimise L(W,RC). For our numerical simulations presented in the next section of this work, we shall use the Levenberg–Marquardt algorithm (cf. [46,47]) for the least squares loss function. The main advantage of this approach is that the loss function helps us to force the peaks of EF wavelets close to the peaks of real data.

The number of log-normal wavelets *N* depends on the data of each population level, since it presents the numbers sub-epidemic. In our numerical simulations, we first try with N=3,5. It would be interesting to estimate *N* before fitting the model. Otherwise, we will need to choose *N* sufficiently large, and redundant wavelets will have very small coefficients and, correspondingly, very little effect.

### 4.2. Data and Smoothing

We will be using the data supplied by the Johns Hopkins University Center [48], noting, however, that almost all data from countries or US federal states are subject to (high) noise. One of the main reason for this is the reporting delay (cf. [49,50]). As explained in [50], *“there will be two main sources of delay in monitoring trends. First of all, there will be a testing delay between the actual date when an individual becomes infected and the date when that individual is ultimately tested. Second, unless test samples are very rapidly processed, there will be a further reporting delay between the date of testing and the date the test results are communicated by the reporting entity.”*

In order to reduce noise, we do smooth out the real data using a (two-sided) moving average method (cf. [51] Chapter 3, cf. [52,53]). A moving average is a time series constructed by taking averages of several sequential values of another time series which is a type of mathematical convolution. In statistics, two-sided moving averages are used to *smooth* a time series in order to estimate or highlight the underlying trend. If we represent the original time series by $x_{1},\ldots ,x_{n}$, then a (simple) two-sided moving average of the time series will be given by

$${\bar{x}}_{i}=\frac{1}{2d+1}\sum_{k=i-d}^{i+d}x_{k}.$$

If the data are showing a periodic fluctuation, moving averages of periods of equal length will eliminate the periodic variations (cf. [51,52]). Observing various population levels indicates that there is periodic fluctuation of 7 days on the data, and thus we will take the average of 7 days

$$\bar{RC}\left(i\right)=\frac{1}{7}\sum_{k=i-3}^{i+3}RC\left(k\right).$$

### 4.3. Projections and Validations for the Czech Republic, France, Germany and Italy

#### 4.3.1. Projections from 25 October 2020

**Figure 3:**
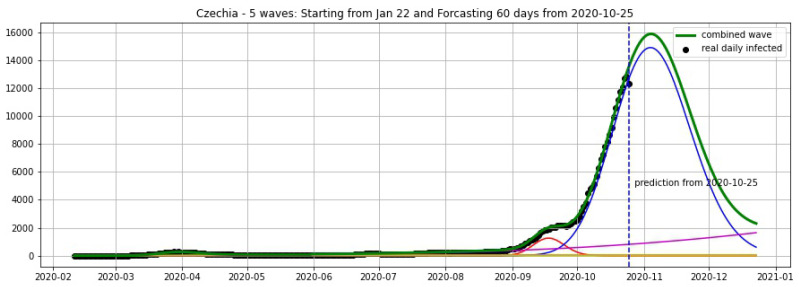
Czechia: fitting and forecasting (green curve) from 25 October with 5 wavelets. The green curve is the combination of other curves which are EF wavelets.

**Figure 4:**
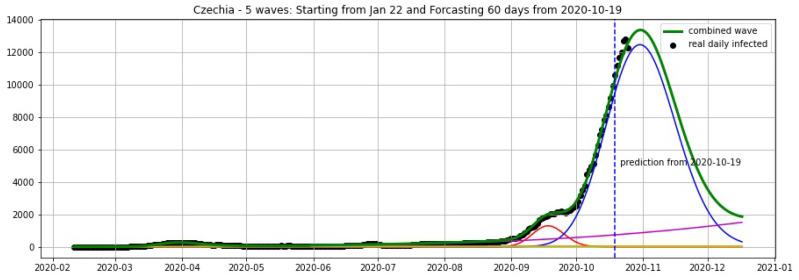
Czechia: fitting and forecasting from 19 October with 5 wavelets. The green curve is the combination of other curves which are EF wavelets.

**Figure 5:**
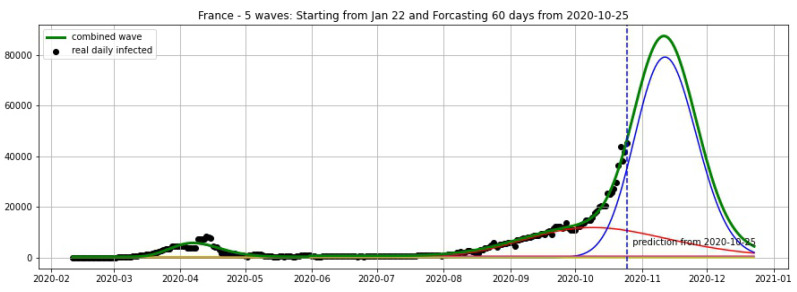
France: fitting and forecasting from 25 October with 5 wavelets. Our model predicts a new wave starting from October 2020.

**Figure 6:**
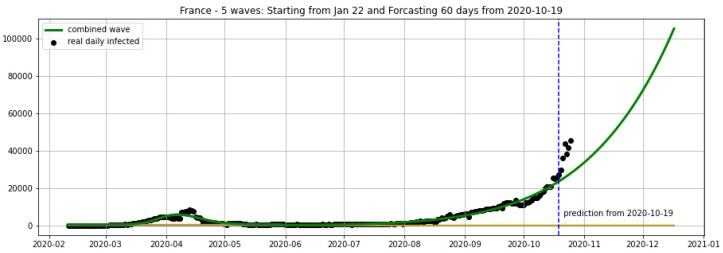
France: fitting and forecasting from 19 October with 5 wavelets. The green curve is the combination of other curves which are EF wavelets.

**Figure 7:**
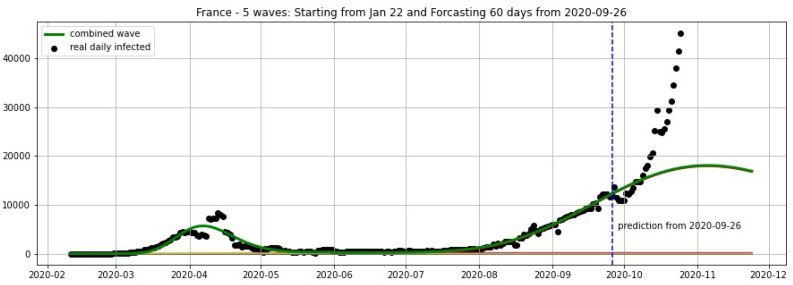
France: fitting and forecasting from 26/09 with 5 wavelets. The green curve is the combination of other curves which are EF wavelets.

**Figure 8:**
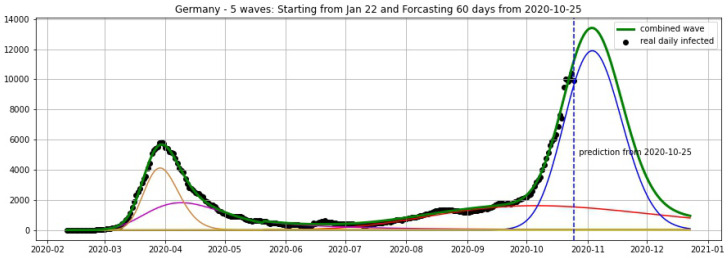
Germany: fitting and forecasting from 25 October with 5 wavelets. The green curve is the combination of other curves which are EF wavelets.

**Figure 9:**
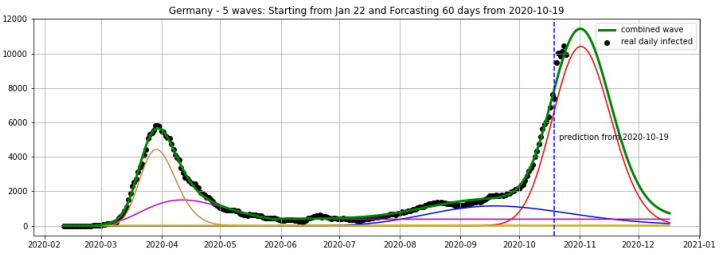
Germany: fitting and forecasting from 19 October with 5 wavelets. The green curve is the combination of other curves which are EF wavelets.

**Figure 10:**
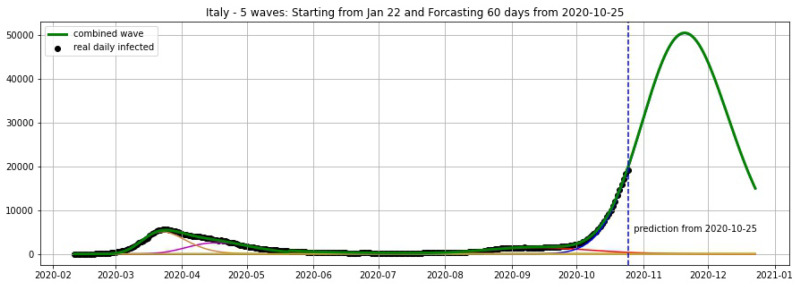
Italy: fitting and forecasting from 25 October with 5 wavelets. The green curve is the combination of other curves which are EF wavelets.

**Figure 11:**
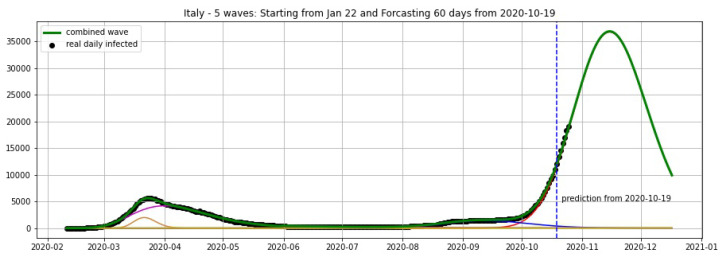
Italy: fitting and forecasting from 19 October with 5 wavelets. The green curve is the combination of other curves which are EF wavelets.

In Figure 3, Figure 4, Figure 5, Figure 6, Figure 7, Figure 8, Figure 9, Figure 10 and Figure 11, the green curve shows the approximate number of daily confirmed new cases and also a possible scenario with a 60-day projection for the Czech Republic (or, in short: Czechia), France, Germany and Italy. Other curves present log-normal EF wavelets where each one can be seen as a sub-epidemic, localised both in time and location. These EF wavelets then give us the nowcasting for the epidemic situation for each population level, i.e., forecasts present sub-epidemics, recent sub-epidemics and the combination of sub-epidemics.

For validation, we use the metric *relative percentage difference:*

(15) $${\mathrm{err}}_{i}=\frac{|y_{i}-{\hat{y}}_{i}|}{y_{i}},$$

where $y_{i}$ is the real data at day *i* smoothed by a 7-days moving average and ${\hat{y}}_{i}$ is the prediction of our model. We fit our model with the data of daily cases until 19 October and keep the last 6 days (20–25 October) for the validation set, then obtain the average error of 4.17% for Czechia, 7.48% for Germany and 3.25% for Italy (see Table 1).

However, we obtain an average error of 32.61% for France (see Figure 6) on the validation set from 20–25 October. We remark here that in some periods of 3 consecutive days the total cases of France remain constant in the Johns Hopkins University data [48], and the total cases are updated by summing up for the day after these 3 days. For example, the periods 9–11 October and 16–18 October show 732,434 and 876,342 total cases, respectively. This makes the daily reported cases equal to zero in some 2 consecutive days. Using a moving average of 7 days we overcome this situation and then use the smoothing data for the projections shown in Figure 5, Figure 6 and Figure 7.

#### 4.3.2. Updated Projections from 9 November 2020

**Figure 12:**
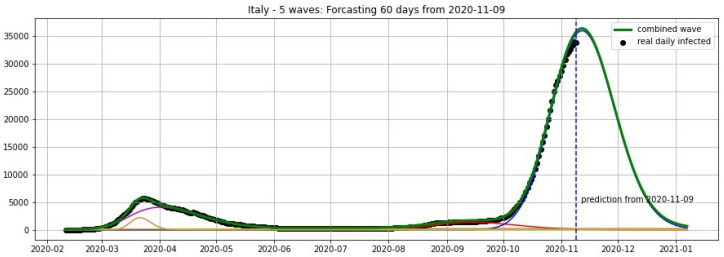
Italy: fitting and forecasting from 9 November with 5 wavelets. The green curve is the combination of other curves which are EF wavelets.

**Figure 13:**
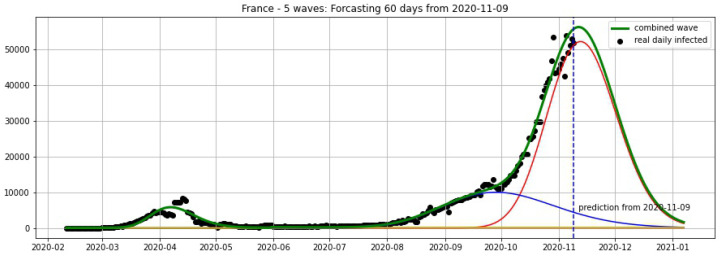
France: fitting and forecasting from 9 November with 5 wavelets. The green curve is the combination of other curves which are EF wavelets.

**Figure 14:**
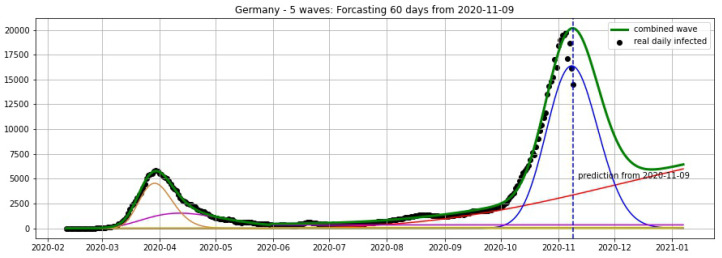
Germany: fitting and forecasting from 9 November with 5 wavelets. The green curve is the combination of other curves which are EF wavelets.

**Figure 15:**
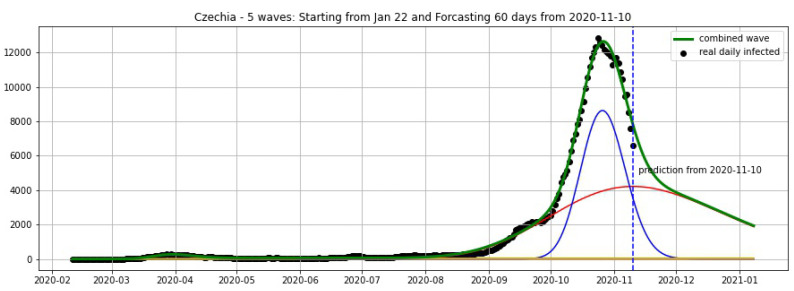
Czechia: fitting and forecasting from 10 November with 5 wavelets. The green curve is the combination of other curves which are EF wavelets.

Figure 12, Figure 13, Figure 14 and Figure 15 show the projections from 9 November 2020.

### 4.4. Projections for Federal States in the United States

**Figure 16:**
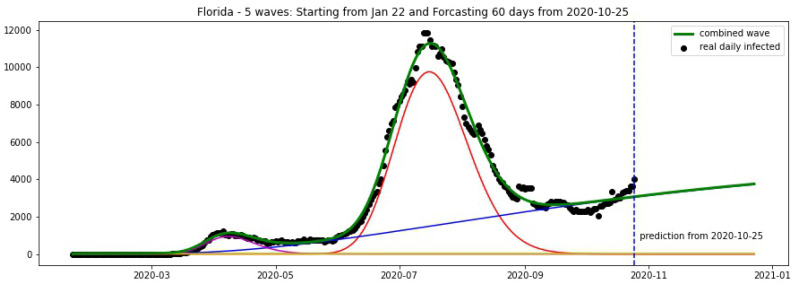
Florida: fitting and forecasting from 25 October. The green curve is the combination of other curves which are EF wavelets.

**Figure 17:**
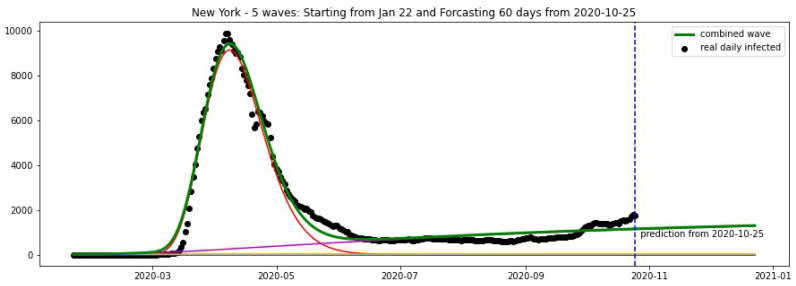
New York: fitting and forecasting from 25 October. The green curve is the combination of other curves which are EF wavelets.

In Figure 16 and Figure 17, the green curve shows the projections for Florida, New York from 25 October 2020.

#### Updated Projections for Florida and New York from 10 November 2020

**Figure 18:**
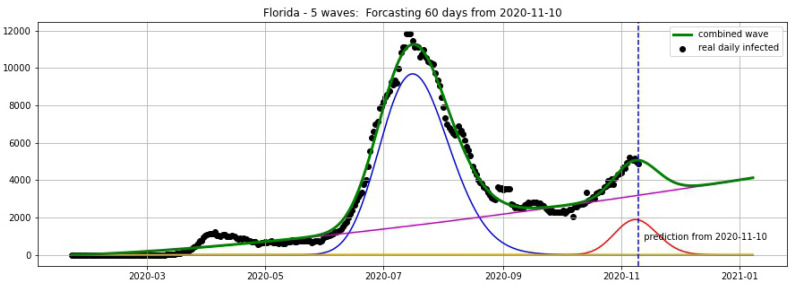
Florida: fitting and forecasting from 10 November 2020. The green curve is the combination of other curves which are EF wavelets.

**Figure 19:**
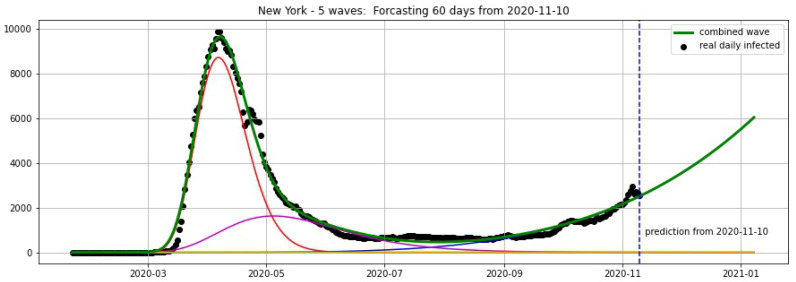
New York: fitting and forecasting from 10 November 2020. The green curve is the combination of other curves which are EF wavelets.

Figure 18 and Figure 19 show the projections for Florida and New York from 10 November 2020.

## 5. Comparing with Other Methods

In this section, we compare our approach to other methods in statistical analysis for forecasting: simple moving average (SMA), autoregressive moving average (ARMA) and autoregressive integrated moving average (ARIMA). We chose two situations: before the first epidemic peak and after the first epidemic peak. We take the average of 7 days for SMA. The parameters for ARMA are p=7,q=7 and the ones for ARIMA are p=7,d=2,q=7.

**Figure 20:**
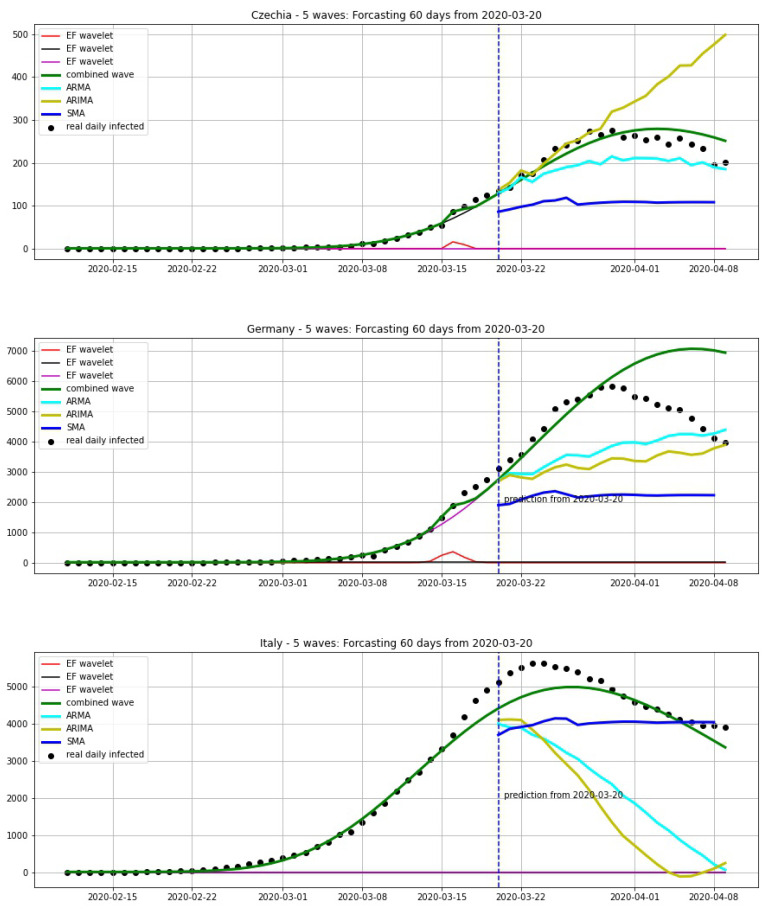
Forecasting 20 days from 30 March, using a wavelet model (green curve) which is combined from EF wavelets, SMA (blue curve), ARMA model (cyan curve) and ARIMA model (yellow curve).

**Figure 21:**
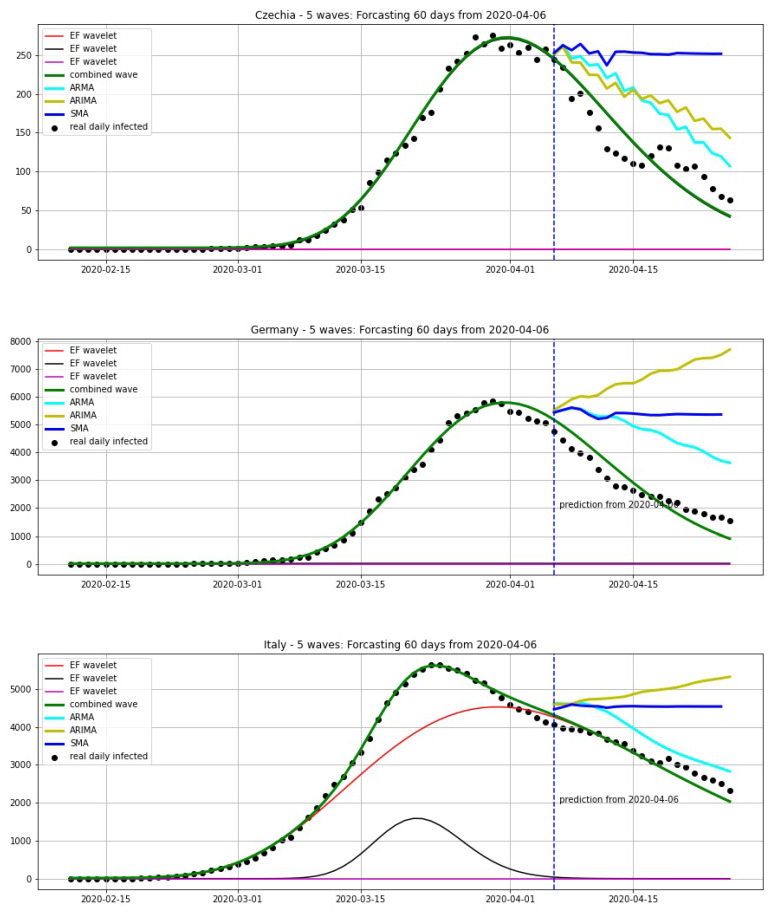
Forecasting 20 days from 06 April, using a wavelet model (green curve) which is combined from EF wavelets, SMA (blue curve), ARMA model (cyan curve) and ARIMA model (yellow curve).

In Figure 20, we compare the forecastings of 20 days from 20 March. We can see that our model can give a good prediction for the peak. In Figure 21, we compare the forecastings of 20 days from 06 April. This shows that our model also gives good results here.

## 6. Conclusions and Outlook

The numerical results in the last section of our paper suggest that our models are actually able to predict the number of daily infected Covid-19 individuals many days ahead in many different countries. In particular, our approach also gives reasonable results for the epidemic situation on population levels by precising sub-epidemics corresponding to EF wavelets.

For solving the curve fitting problem in our model selection, we only have to use relatively few parameters. The model can be seen as a neural network containing only one hidden layer with a log-normal function activation, entailing that we do not have to deal with overfitting problems and that the estimation error of our model is low [54]

Our method for modelling the number of daily reported cases of infectious individuals also applies to other epidemics characteristics, e.g., to the number of active cases, and thus is also important for health care system decisions.

In future work, we will present refinements of our approach as well as refinements of the curve fitting techniques employed here. We will also extend our approach based on the epidemic-fitted wavelet approach to situations where EF wavelets are multivariate functions of time variables, measurement levels, or other variables such as death rate, recovery rate, etc. 

## Figures and Tables

**Table 1:** Prediction by log-normal wavelet model for Czechia, Germany, Italy from 20 October to 25 October.

		Czechia		
**Day**	**Real Data**	**Smoothing**	**Prediction**	**Error**
20 October	11,984	11,173	10,730	3.96%
21 October	14,969	11,710	11,161	4.68%
22 October	14,150	12,030	11,564	3.87%
23 October	15,258	12,689	11,934	5.95%
24 October	12,474	12,830	12,269	4.37%
25 October	7300	12,295	12,564	2.18%
		**Germany**		
**Day**	**Real Data**	**Smoothing**	**Prediction**	**Error**
20 October	8523	9472	8346	11.88%
21 October	12,331	10,019	8763	12.53%
22 October	5952	9861	9164	7.06%
23 October	22,236	10,105	9545	5.54%
24 October	8688	10,421	9902	4.98%
25 October	2900	9944	10,231	2.88%
		**Italy**		
**Day**	**Real Data**	**Smoothing**	**Prediction**	**Error**
20 October	10,871	13,322	13,000	2.41%
21 October	15,199	14,567	14,080	3.34%
22 October	16,078	15,934	15,203	4.58%
23 October	19,143	17,034	16,364	3.93%
24 October	19,640	18,266	17,557	3.88%
25 October	21,273	19,033	18,777	1.34%
